# Upadacitinib in refractory acquired reactive perforating collagenosis with atopic dermatitis and chronic kidney disease: a case report

**DOI:** 10.3389/fmed.2026.1924464

**Published:** 2026-10-07

**Authors:** Qing Wang, Maolin Yao, Lvyao Wang, Xiao Qiu, Hao Lu, Xiangyu Hu, Yuanfang Wan, Juanjuan Liu

**Affiliations:** 1 Department of Dermatology, Chongqing Hospital of Traditional Chinese Medicine, Chongqing, China; 2 College of Traditional Chinese Medicine, Chongqing Medical University, Chongqing, China; 3 Department of Dermatology, Suining Municipal Hospital of Traditional Chinese Medicine, Suining, China

**Keywords:** acquired reactive perforating collagenosis, atopic dermatitis, chronic kidney disease, janus kinase inhibitor, upadacitinib

## Abstract

**Background:**

Acquired reactive perforating collagenosis (ARPC) is a rare perforating dermatosis characterized by transepidermal elimination of degenerated collagen fibers. It frequently develops secondary to systemic comorbidities, including diabetes mellitus, chronic kidney disease (CKD), and atopic dermatitis (AD), which limit therapeutic options and contribute to unpredictable outcomes. Here, we report a case of ARPC with concurrent AD in a patient with type 2 diabetes mellitus and stage 4 CKD, who experienced acute paradoxical exacerbation within 24 h of dupilumab initiation and achieved sustained remission with upadacitinib.

**Case summary:**

A 72-year-old male presented with a 5-year history of recurrent generalized pruritus, erythema, papules, and nodules. Despite treatment with glucocorticoids and anti-allergic medications, the skin lesions remained poorly controlled. Histopathological examination revealed transepidermal elimination of collagen fibers through the ulcer bed into the underlying dermis. Verhoeff-van Gieson staining demonstrated collagen fibers traversing the ulceration. The patient was diagnosed with ARPC. He had poorly controlled comorbidities, including AD, type 2 diabetes mellitus, and stage 4 CKD. Dupilumab was administered as a single 600 mg dose but was discontinued due to paradoxical worsening within 24 h. Following two years of recurrent relapses despite systemic corticosteroids, antihistamines, and thalidomide, oral upadacitinib 15 mg once daily was initiated, with marked resolution of skin lesions and pruritus. After 9 months of treatment, upadacitinib was discontinued, and the patient remained in stable remission at 1-year follow-up. No adverse effects were observed, and renal function remained stable throughout treatment.

**Conclusion:**

This case suggests that upadacitinib is a promising salvage therapy for refractory ARPC, particularly after failure of Th2-targeted biologics. Its favorable safety profile at the standard dose without adjustment in stage 4 CKD supports its use in patients with complex comorbidities.

## Introduction

Acquired reactive perforating collagenosis (ARPC) is a rare perforating dermatosis characterized by transepidermal elimination of degenerated collagen fibers. Clinically, it presents with pruritic papules and nodules, often with central ulceration, crusting, and umbilication, predominantly affecting the trunk and extremities. Although its exact pathogenesis remains incompletely understood, repetitive mechanical irritation and microangiopathy are thought to contribute to collagen degeneration and impaired clearance of metabolic byproducts (1, 2). ARPC frequently develops secondary to systemic comorbidities, particularly diabetes mellitus and chronic kidney disease (CKD) (3). Notably, ARPC and atopic dermatitis (AD) share common Th2-driven inflammatory pathways (4). Current treatments, including narrowband ultraviolet B phototherapy, antihistamines, and systemic agents such as allopurinol or retinoids, have shown variable efficacy and are often limited by concurrent systemic comorbidities. Biologics such as dupilumab and Janus kinase (JAK) inhibitors have shown efficacy in refractory ARPC (3, 5). Here, we report a case of refractory ARPC with AD, type 2 diabetes mellitus, and stage 4 CKD, in which dupilumab therapy failed due to acute exacerbation within 24 h, whereas upadacitinib, initiated approximately two years later, achieved sustained remission.

## Case report

A 72-year-old male patient presented with a 5-year history of generalized erythema, papules, nodules, and severe pruritus. He had been diagnosed with AD two years prior. Family history of atopic diseases was unavailable. He had previously received multiple systemic agents, including compound glycyrrhizin, tripterygium wilfordii, tofacitinib, epinastine, and olopatadine, as well as topical neomycin sulfate, human epidermal growth factor, and betamethasone propionate, without adequate response. He also had a 6-year history of type 2 diabetes mellitus and stage 4 CKD, both poorly controlled and managed conservatively without hemodialysis or peritoneal dialysis. Physical examination revealed widespread firm erythema, papules, and nodules ranging from rice-grain to fingernail-plate size on the scalp, trunk, and extremities. Some lesions showed central ulceration with yellow-brown crusts, and several nodules exhibited mild surrounding erythema, a rough keratotic surface, and central umbilication (Figures 1A–F). The SCORing Atopic Dermatitis (SCORAD) score was 48.8 at presentation.

**Figure 1:**
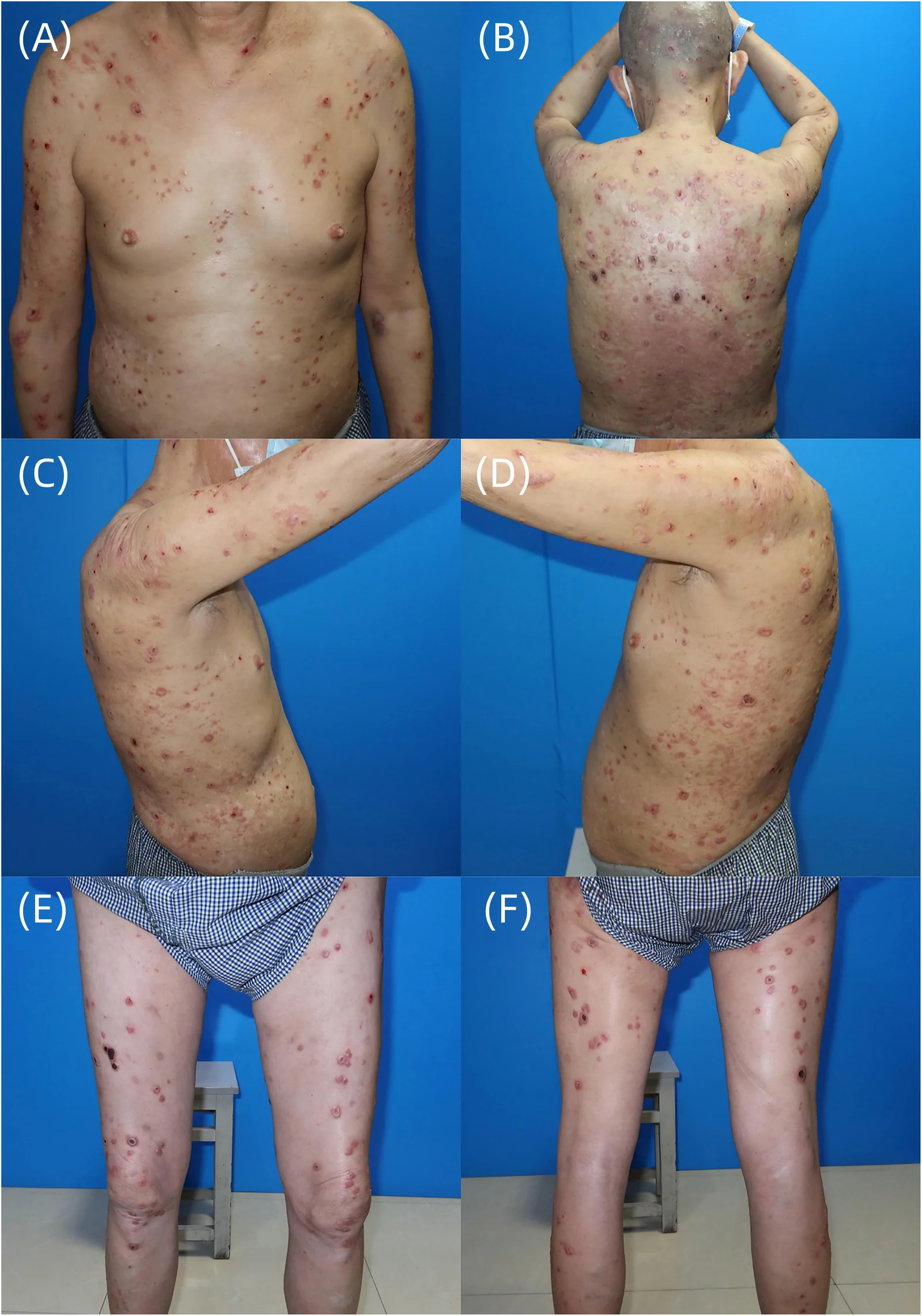
**(A–F)** prior to upadacitinib treatment, the patient presented with generalized erythema, papules and nodules.

During hospitalization, a series of laboratory examinations revealed abnormal findings: HbA1c was 11.6%, random capillary blood glucose was 33.3 mmol/L, and fasting blood glucose was 35.23 mmol/L. Complete blood count demonstrated leukocytosis (16.20 × 10⁹/L), anemia (hemoglobin 94 g/L), eosinophilia (1.90 × 10⁹/L, 15.9%), and thrombocytosis (403 × 10⁹/L). Inflammatory markers were elevated, including C-reactive protein (132.11 mg/L) and fibrinogen (6.430 g/L). Biochemical analysis revealed hypoalbuminemia (29.7 g/L), azotemia (BUN 21.21 mmol/L), hyperuricemia (498 μmol/L), elevated creatinine (298.43 μmol/L), and elevated cystatin C (2.77 mg/L). Twenty-four-hour urine collection indicated proteinuria (355.4 mg/L; total 746.34 mg/24 h), urine volume of 2.10 L, and reduced creatinine clearance (24.3 mL/min). Cytokine assays showed elevated interleukin (IL)-4 (8.54 pg/mL; reference 0–2.80 pg/mL), IL-6 (19.13 pg/mL; reference 0–5.30 pg/mL). Total serum IgE was 581.61 IU/mL. Allergen screening detected grade 1 sensitization to crab (0.60 kU/L). Interferon-gamma release assay (IGRA) was negative, and HIV and syphilis serology were negative. Flow cytometry revealed a decreased absolute count of CD8+ T cells (195/*μ*L), a CD4+ T cell percentage of 68.37%, a CD4/CD8 ratio of 4.02, and an absolute lymphocyte count of 700/μL, consistent with relative immunosuppression. Non-contrast chest CT identified a small nodule in the anterior segment of the right upper lobe and several fibrocalcified foci in both lungs.

Histopathological examination revealed epidermal hyperplasia, focal necrosis, erosion, and ulceration. Transepidermal elimination of collagen fibers was evident through the ulcer bed into the underlying dermis. The superficial dermis contained patchy perivascular infiltrates composed of lymphocytes, histiocytes, and numerous plasma cells (Figure 2A). Verhoeff-van Gieson staining demonstrated collagen fibers traversing the ulceration (Figure 2B).

**Figure 2:**
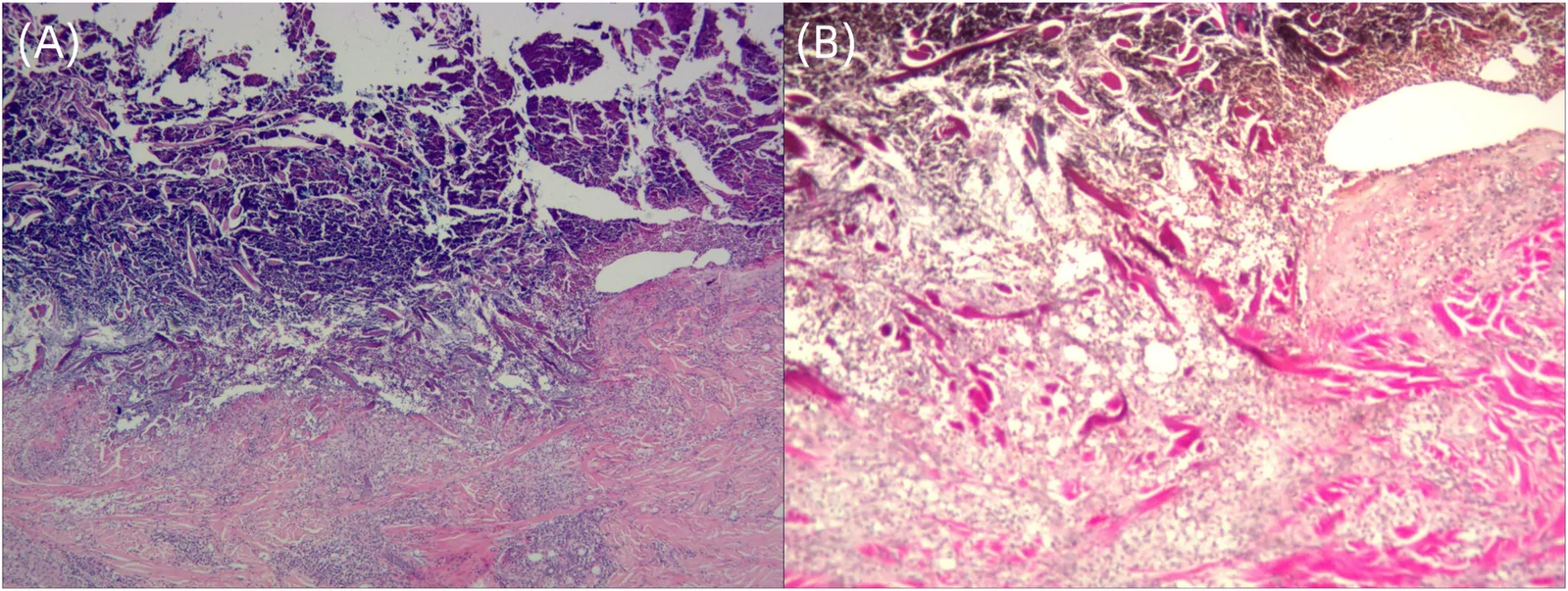
Histopathological picture **(A)** and verhoeff-van gieson staining **(B)** of patient.

Based on these findings, the patient was diagnosed with ARPC, concurrent AD [based on Chinese Zhang's criteria (6): over six months of symmetrical eczema, total IgE of 581.61 IU/mL, peripheral eosinophilia at 1.9 × 10⁹/L, and a personal history of allergic rhinitis], type 2 diabetes mellitus, and stage 4 CKD. Because of the patient's elevated eosinophil count, high SCORAD score, multiple comorbidities, and impaired renal function, conventional anti-inflammatory and immunosuppressive agents were largely contraindicated. Consequently, dupilumab was administered as a single 600-mg initial dose. Within 24 h, the patient developed extensive oedematous erythema and bright-red papules on the face, neck, trunk, and both upper extremities, with severe pruritus and acute worsening of skin lesions (Figures 3A,B). A paradoxical reaction to dupilumab was suspected. Intramuscular dexamethasone 5 mg once daily was given for five days, followed by oral methylprednisolone 8 mg daily, tapered to 4 mg daily. Given the paradoxical reaction and relative immunosuppression evidenced by T-cell subset testing, dupilumab was permanently discontinued. Other therapeutic measures included insulin aspart and insulin glargine for glycaemic control, and continuation of medications for CKD management.

**Figure 3:**
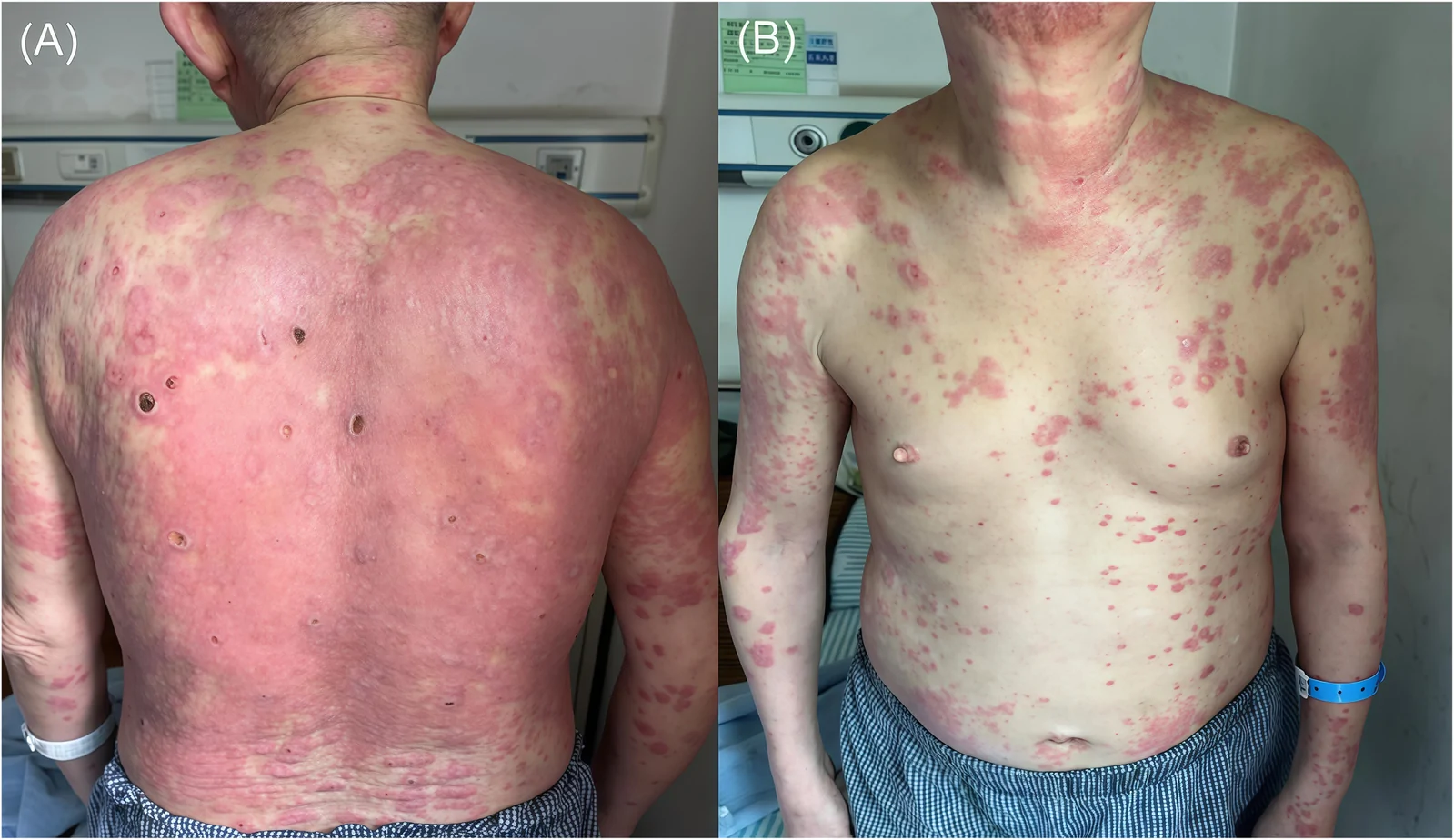
**(A**, **B)** clinical picture of patient after 24 h of treatment with dupilumab.

During the subsequent two years, the patient was hospitalized twice for recurrent ARPC and received interim therapies including systemic corticosteroids, epinastine, loratadine, thalidomide, and topical halometasone, neomycin gel, and fusidic acid. These interventions yielded limited benefit, with consistent relapse upon glucocorticoid tapering. Due to refractory ARPC and severe renal insufficiency, oral upadacitinib 15 mg once daily was started approximately two years after dupilumab discontinuation. Prior to upadacitinib initiation, pre-treatment screening comprising hepatic function tests, lipid profile, C-reactive protein, repeat IGRA, and repeat chest computed tomography revealed no significant abnormalities. No contraindications to JAK inhibitor therapy were identified. At the time of upadacitinib initiation, laboratory examination revealed an eosinophil count of 0.56 × 10⁹/L, an estimated creatinine clearance of 21.39 mL/min, a total IgE of 654.15 IU/mL, and a fasting blood glucose of 10.16 mmol/L. After 3 weeks, pruritus improved significantly (PP-NRS 9 to 2). Most papules and nodules flattened, ulcers healed, and the SCORAD score decreased from 48.8 to 29. After 8 weeks, skin lesions had nearly resolved, with only post-inflammatory hyperpigmentation and atrophic scars remaining (Figures 4A–D). The SCORAD score further decreased to 8, and total serum IgE normalized. No recurrence of pruritus or severe adverse events was observed, and liver and renal function were monitored regularly during treatment. The patient continued standard therapies for diabetes mellitus and CKD; creatinine clearance remained stable at 15.5–23 mL/min, with fasting blood glucose ranging from 25 to 30 mmol/L. After 9 months, all papules and nodules had resolved, and pruritus was nearly eliminated. Following clinical remission and at the patient's request, upadacitinib was discontinued. At 1-year follow-up, the patient remained stable without recurrence.

**Figure 4:**
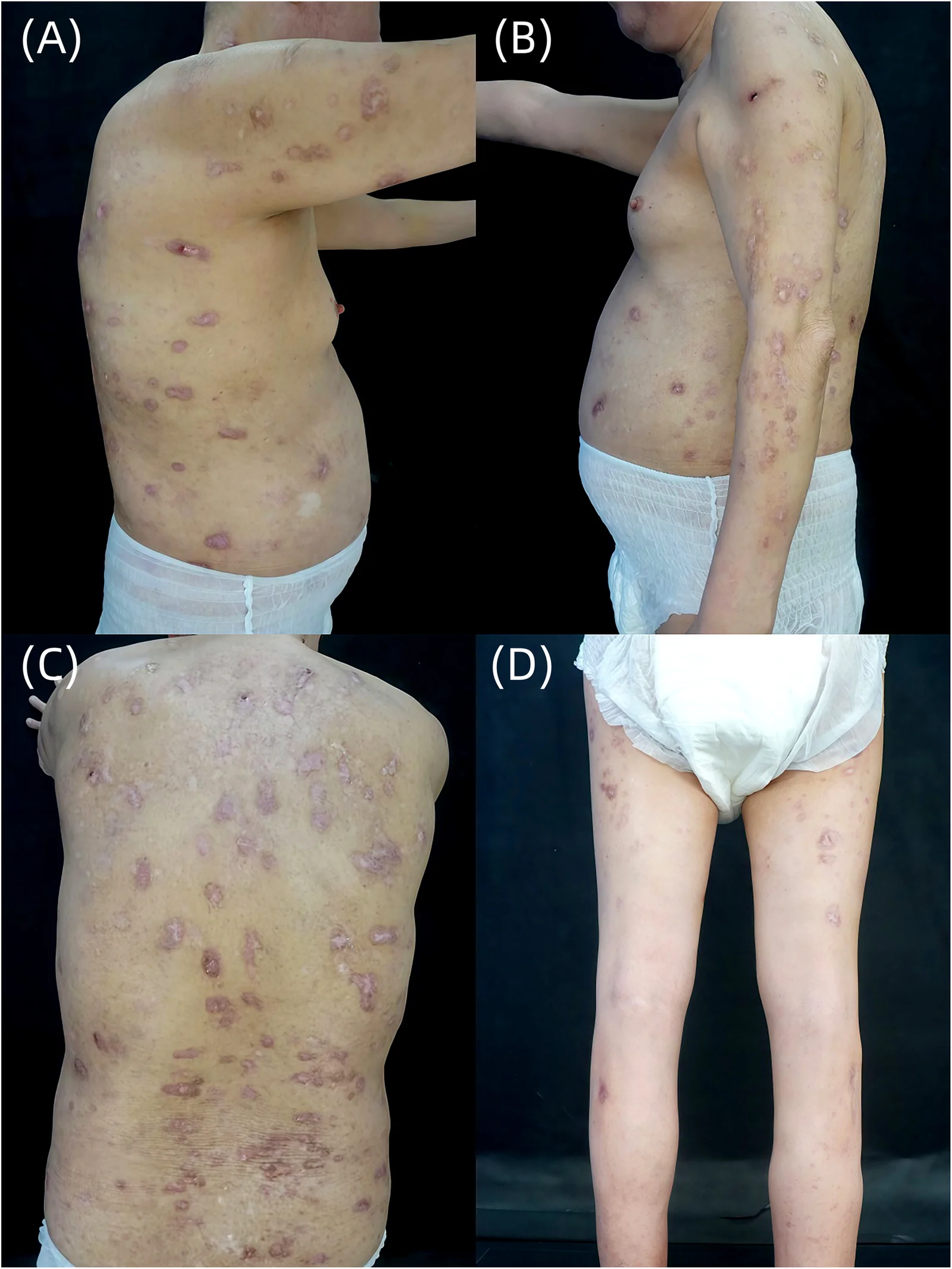
**(A-D)** At 8 weeks after upadacitinib treatment, the patient showed residual hyperpigmented lesions and atrophic scars on the trunk and extremities.

## Discussion

Severe pruritus in AD leads to chronic scratching, resulting in aberrant wound repair and disordered collagen metabolism that underlies the pathogenesis of ARPC (7). Th2-type inflammation, particularly IL-4 and IL-13, drives IgE production and eosinophilic infiltration in both conditions, providing a rationale for dupilumab (anti-IL-4R*α*) and JAK inhibitors (4, 8). Nevertheless, dupilumab did not provide therapeutic benefit in this patient and instead induced paradoxical worsening. Although dupilumab selectively suppresses Th2 inflammation by blocking IL-4R*α* (8), recent reports have noted its potential to induce paradoxical psoriasiform or rosacea-like reactions (9). Several factors contributed to treatment failure in this case. While IL-4/IL-13 drive pruritus in AD, metabolic stress in this patient also activated non-Th2 pathways including JAK-dependent IL-31, Th17-related cytokines, and TGF-*β*-mediated fibrotic remodeling, rendering IL-4/IL-13 blockade alone insufficient. Flow cytometry revealed relative immunosuppression, which may have predisposed the patient to an atypical response. The persistent itch-scratch cycle also continued despite targeted Th2 inhibition, perpetuating new lesion formation.

However, in patients with concurrent metabolic disorders, Th2-targeted therapy alone may be insufficient. In this patient, the combination of type 2 diabetes mellitus and severe CKD created a significant metabolic burden. Accumulation of advanced glycation end products (AGEs) and uremic toxins contributes to dermal microangiopathy, collagen degeneration, and neuronal sensitization, which worsen the itch-scratch cycle and promote transepidermal collagen elimination (10), while uremic toxins also maintain a systemic pro-inflammatory state (11). These metabolic disturbances and Th2-driven inflammation were not independent processes but formed a self-sustaining pathogenic loop. Furthermore, severe renal insufficiency restricted the use of conventional immunosuppressants; methotrexate was contraindicated due to renal excretion, and cyclosporin posed nephrotoxic risks that may further impair renal function. Therefore, effective therapy in this population must address both immune dysregulation and the itch-scratch cycle, with proven safety in advanced CKD.

Upadacitinib, a highly selective JAK1 inhibitor, effectively suppresses JAK1-dependent signaling involving IL-4/IL-13, IL-31, IL-17, and several pruritogenic mediators (12, 13). Unlike dupilumab, which targets only the Th2 pathway, and less selective JAK inhibitors, upadacitinib inhibits both Th2-dominant and non-Th2-dominant pathogenic pathways in ARPC. This broad mechanism aligns with the complex cytokine network characteristic of ARPC (12). Notably, the patient had previously received tofacitinib, a non-selective JAK1/3 inhibitor, without adequate response. His subsequent robust response to the highly selective JAK1 inhibitor upadacitinib suggests that JAK1 selectivity may be a critical determinant of therapeutic efficacy in refractory ARPC. These pharmacological properties likely account for the positive therapeutic response to upadacitinib after dupilumab treatment failure in this patient. In a randomized clinical trial, upadacitinib demonstrated favorable tolerability and greater efficacy compared to dupilumab at Week 16 (13). Clinically, upadacitinib markedly reduced pruritus, interrupted the itch-scratch cycle within 3 weeks, and promoted healing of ulcerative lesions. Pharmacokinetic data indicate that the area under the concentration-time curve from zero to infinity (AUCinf) for 15 mg once-daily upadacitinib increases by 18%, 33%, and 44% in patients with mild, moderate, and severe renal impairment, respectively, compared with subjects with normal renal function. Given the limited impact on systemic drug exposure, dose adjustment for renal dysfunction is not recommended (14).

This case differs from previously published reports in several respects. Most reported ARPC cases treated with JAK inhibitors such as upadacitinib (5, 15), baricitinib (16) and tofacitinib (17) occurred in patients without severe renal impairment. In contrast, our patient exhibited ARPC against a background of both type 2 diabetes mellitus and stage 4 CKD, in whom metabolic toxins and microvascular injuries served as ongoing pathogenic factors. Comparison with published cases indicates that upadacitinib can be highly effective even in patients with substantial metabolic burden and dupilumab resistance, thereby adding to real-world evidence for this refractory ARPC phenotype. Several key insights can be drawn from this case. This case demonstrates that JAK inhibitors can serve as effective rescue therapy when Th2-targeted biologics are insufficiently effective or provoke paradoxical reactions, as illustrated by the successful transition to upadacitinib after dupilumab failure. It also illustrates the efficacy and tolerability of upadacitinib in patients with severe renal impairment. As such patients were largely excluded from pivotal AD clinical trials, this observation helps address that evidence gap. Moreover, remission was sustained for an extended period after discontinuation at nine months, in contrast to published reports of relapse after baricitinib withdrawal (16). This suggests that treatment duration should be individualized for certain ARPC patients and that close follow-up is essential after treatment cessation. Finally, recent literature has described secondary ARPC developing after upadacitinib treatment for AD (18), underscoring the complex and context-dependent immunomodulatory effects of JAK inhibitors. The same agent may yield divergent outcomes in different clinical scenarios, potentially due to comorbidities, treatment timing, and individual immune variability. Further investigation is warranted.

## Conclusion

This case suggests that, in the context of ARPC with metabolic disorders and Th2 inflammation, upadacitinib offers selective JAK1 inhibition, modulates immune dysregulation, and disrupts the itch-scratch cycle. Additionally, this case demonstrates a favorable safety profile of upadacitinib in a patient with stage 4 CKD. However, these observations are limited to a single case. Further prospective studies are required to establish the optimal use of upadacitinib for refractory ARPC, particularly regarding treatment duration and discontinuation strategies.

## Data Availability

The original contributions presented in the study are included in the article/Supplementary Material, further inquiries can be directed to the corresponding author/s.

## References

[1] Thiele-Ochel S Schneider LA Reinhold K Hunzelmann N Krieg T Scharffetter-Kochanek K. Acquired perforating collagenosis: is it due to damage by scratching? Br J Dermatol. (2001) 145(1):173–4. 10.1046/j.1365-2133.2001.04309.x 11453934

[2] García-Malinis AJ Del Valle Sánchez E Sánchez-Salas MP Del Prado E Coscojuela C Gilaberte Y. Acquired perforating dermatosis: clinicopathological study of 31 cases, emphasizing pathogenesis and treatment. J Eur Acad Dermatol Venereol. (2017) 31(10):1757–63. 10.1111/jdv.14220 28300323

[3] Junghetu MA Tutunaru CV Ianoși SL Georgescu CV Orzan OA. Acquired reactive perforating collagenosis-A rare entity occurring within common disorders: a systematic review and our personal experience. J Clin Med. (2026) 15(1):391. 10.3390/jcm15010391. 41517640 PMC12787290

[4] Liu B Wu Y Wu X Zhong X Xue R Zhang Z. Dupilumab improve acquired reactive perforating collagenosis characterized by type 2 inflammation. Front Immunol. (2023) 14:1240262. 10.3389/fimmu.2023.1240262 37638036 PMC10449391

[5] Chen J Deng T Li M Lian C. Efficacy of upadacitinib in the treatment of acquired reactive perforating collagenosis in a patient with type 1 diabetes: a case report and A literature review. Clin Cosmet Investig Dermatol. (2025) 18:2489–94. 10.2147/CCID.S549157 41050714 PMC12493097

[6] Zhao W Li Y. A comparative study of three diagnostic criteria for atopic dermatitis. Diagn Ther J Dermato-Venereol. (2023) 30(2):120–4. 10.3969/j.issn.1674-8468.2023.02.005

[7] Radi G Campanti A Diotallevi F Martina E Marani A Offidani A. A systematic review of atopic dermatitis: the intriguing journey starting from physiopathology to treatment, from laboratory bench to bedside. Biomedicines. (2022) 10(11):2700. 10.3390/biomedicines10112700 36359220 PMC9688004

[8] Gori N De Luca E Chiricozzi A Sfregola S Stefani AD Peris K. Successful use of dupilumab in the treatment of acquired perforating dermatosis associated with atopic dermatitis. Case Rep Dermatol Med. (2024) 2024:6265608. 10.1155/2024/6265608 38939121 PMC11208807

[9] Camela E Giampetruzzi AR De Pità O Pallotta S Russo F. Dupilumab in real-life settings: a review of adverse events and their pathogenesis. Expert Opin Drug Saf. (2024) 23(4):439–47. 10.1080/14740338.2024.2326480 38470213

[10] Khalid M Petroianu G Adem A. Advanced glycation end products and diabetes mellitus: mechanisms and perspectives. Biomolecules. (2022) 12(4):542. 10.3390/biom12040542 35454131 PMC9030615

[11] Espi M Koppe L Fouque D Thaunat O. Chronic kidney disease-associated immune dysfunctions: impact of protein-bound uremic retention solutes on immune cells. Toxins (Basel). (2020) 12(5):300. 10.3390/toxins12050300 32384617 PMC7291164

[12] Guttman-Yassky E Thaçi D Pangan AL Hong HC- Papp KA Reich K. Upadacitinib in adults with moderate to severe atopic dermatitis: 16-week results from a randomized, placebo-controlled trial. J Allergy Clin Immunol. (2020) 145(3):877–84. 10.1016/j.jaci.2019.11.025 31786154

[13] Blauvelt A Teixeira HD Simpson EL Costanzo A De Bruin-Weller M Barbarot S. Efficacy and safety of upadacitinib vs dupilumab in adults with moderate-to-severe atopic dermatitis: a randomized clinical trial. JAMA Dermatol. (2021) 157(9):1047–55. 10.1001/jamadermatol.2021.3023 34347860 PMC8340015

[14] Mohamed MF Trueman S Feng T Anderson J Marbury TC Othman AA. Characterization of the effect of renal impairment on upadacitinib pharmacokinetics. J Clin Pharmacol. (2019) 59(6):856–62. 10.1002/jcph.1375 30633369 PMC6590375

[15] Ding W Wang Y Song L Zhou N. A potential new treatment with upadacitinib for acquired reactive perforating collagenosis. JAAD Case Rep. (2024) 48:112–4. 10.1016/j.jdcr.2024.04.017 38813063 PMC11134568

[16] Zheng J Ding Y Chen Y Shi Y Gao Y. Effectiveness of baricitinib in acquired reactive perforating collagenosis: a case report. Front Immunol. (2024) 15:1388274. 10.3389/fimmu.2024.1388274 39076971 PMC11284048

[17] Yuan R Zhou G Liu H. Tofacitinib for treatment of acquired reactive perforating collagenosis. JAMA Dermatol. (2025) 161:446. 10.1001/jamadermatol.2024.6280 40009392

[18] Morrison E Low SE Ngan K. A unique case of reactive perforating collagenosis secondary to upadacitinib treatment of severe atopic eczema. Clin Exp Dermatol. (2025) 50(7):1452–3. 10.1093/ced/llaf122 40080678

